# Recent advances in nanomaterial-modified electrical platforms for the detection of dopamine in living cells

**DOI:** 10.1186/s40580-020-00250-7

**Published:** 2020-12-22

**Authors:** Yeon-Woo Cho, Joon-Ha Park, Kwang-Ho Lee, Taek Lee, Zhengtang Luo, Tae-Hyung Kim

**Affiliations:** 1 School of Integrative Engineering, Chung-Ang University, Seoul, 06974 Republic of Korea; 2 Department of Chemical Engineering, Kwangwoon University, Wolgye-dong, Nowon-gu, 01899 Seoul, Republic of Korea; 3 Department of Chemical and Biological Engineering, Hong Kong University of Science and Technology, Kowloon, 999077 Hong Kong China; 4 Integrative Research Center for Two-dimensional Functional Materials, Institute of Interdisciplinary Convergence Research, Chung Ang University, Seoul, 06974 Republic of Korea

**Keywords:** Conductive polymers, Dopamine, Electrical detection, Gold nanostructure, Graphene

## Abstract

Dopamine is a key neurotransmitter that plays essential roles in the central nervous system, including motor control, motivation, arousal, and reward. Thus, abnormal levels of dopamine directly cause several neurological diseases, including depressive disorders, addiction, and Parkinson’s disease (PD). To develop a new technology to treat such diseases and disorders, especially PD, which is currently incurable, dopamine release from living cells intended for transplantation or drug screening must be precisely monitored and assessed. Owing to the advantages of miniaturisation and rapid detection, numerous electrical techniques have been reported, mostly in combination with various nanomaterials possessing specific nanoscale geometries. This review highlights recent advances in electrical biosensors for dopamine detection, with a particular focus on the use of various nanomaterials (e.g., carbon-based materials, hybrid gold nanostructures, metal oxides, and conductive polymers) on electrode surfaces to improve both sensor performance and biocompatibility. We conclude that this review will accelerate the development of electrical biosensors intended for the precise detection of metabolite release from living cells, which will ultimately lead to advances in therapeutic materials and techniques to cure various neurodegenerative disorders.

## Introduction

As reported by the Global Burden of Disease Study 2016, neurodegenerative disorders, a group of diseases mainly caused by neurodegeneration, have emerged as highly serious issues [1–3]. Specifically, neurodegenerative diseases caused approximately 9,000,000 deaths, which amounted to 16.5% of total worldwide deaths in 2016. Moreover, as global life expectancy has been increasing, it is expected that deaths caused by neurodegenerative disorders will also increase, considering that aging is the main factor for the disease [4, 5]. Most neurodegenerative disorders, such as Parkinson’s disease (PD), Alzheimer’s disease, and Huntington’s disease (HD) are indeed related to protein aggregation due to misfolding [6–9]. Aggregates and other downstream biomolecules have been regarded as pathological markers for neurodegenerative disorders [10–12].

Dopamine (DA) is an essential neurotransmitter in the nervous system responsible for various activities, including motor control, motivation, arousal, and reward, and it is one of the most critical biomolecules in the human body [13]. Unfortunately, protein aggregation induces dysfunction and mass loss of dopaminergic neurons, resulting in abnormal DA levels in the human blood, which ultimately leads to severe neurodegenerative disorders (e.g., HD and PD) [14, 15]. Based on the pathological mechanism, two physiological strategies have been proposed to cure these diseases: (i) the improvement or recovery of DA release as one of the main cellular functions of dopaminergic neurons, and (ii) *in vitro* generation of mature dopaminergic neurons for in vivo transplantation. Specifically, such strategies involve drug-screening techniques capable of analysing the effects of specific drugs on DA release by dopaminergic neurons and regeneration of damaged dopaminergic neurons. For these reasons, sensing techniques that enable the precise detection of DA release from living dopaminergic neurons are essential for discovering useful therapeutic materials and techniques to cure the diseases.

Bioelectronic techniques, including electrochemical (EC), electrochemiluminescence (ECL), and field-effect transistors (FETs), have been widely investigated as DA sensors because they can achieve miniaturisation of sensing platforms with rapid detection times [16–19]. EC-based biosensing techniques have been intensively studied because DA itself is a redox-active molecule and can be readily detected without using any bioligands, enzymes, or reporter probes [20–24]. However, EC methods have several limitations, especially the selectivity and sensitivity toward DA when co-existing with other interfering biomolecules such as glucose, ascorbic acid, uric acid, and other catecholamines (e.g., epinephrine, norepinephrine, and L-DOPA).

Nanomaterials have distinct physicochemical properties, and have been widely used to improve the sensing performance of EC techniques [25–30]. Nanomaterials possess a high electroactive surface area and facilitate electron transfer between the target molecule and electrode surface, which ultimately improves the sensitivity of the electrical sensing platform. Other advantages, including high electrical conductivity, electrical mobility, and electrocatalytic properties, have also been reported to enhance both selectivity and sensitivity toward DA detection [31–34]. Specifically, carbon-based materials, including carbon nanotubes (CNTs) and graphene derivatives, can absorb DA via π-π stacking and enhance DA-specific signals in combination with their excellent electrical properties [35–41]. Gold nanoparticles and nanostructures are ideal materials for DA sensing because they possess exceptional electrical conductivity and electrocatalytic activity [42]. Gold is preferred for modification of sensor platforms owing to its highly stable nature in cell cultivation conditions requiring high temperatures (~ 37 °C) and high humidity (~ 100%) as well as its excellent biocompatibility toward animal cells [43, 44]. Other materials, including metal oxides and conductive polymers, are also known to be biocompatible and can enhance DA-specific electrical signals via specific chemical or physical interactions with DA [45–50].

**Fig. 1:**
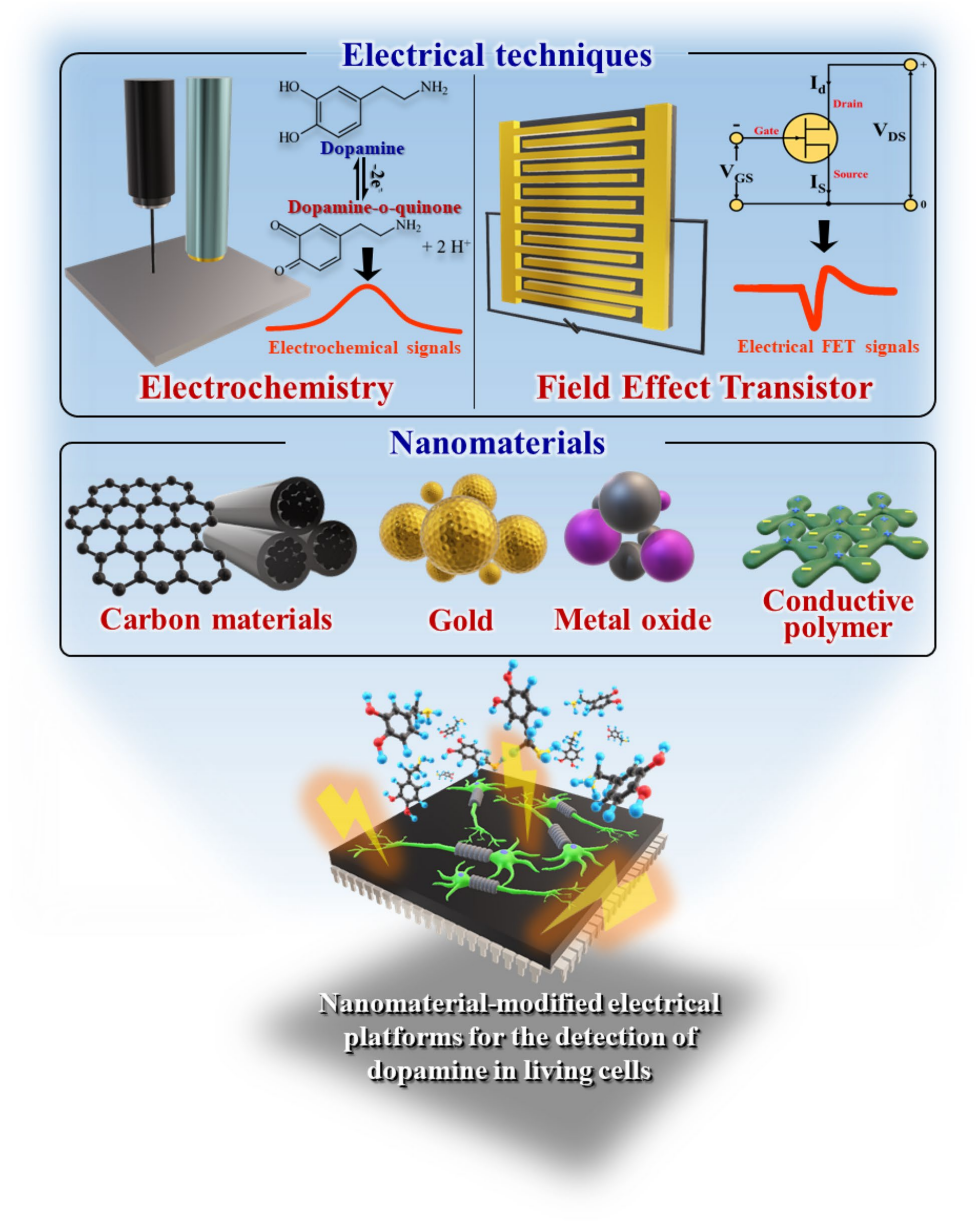
Schematic illustration representing recent advances in nanomaterial-modified EC platforms for the detection of dopamine from living cells

Based on this trend in the development of biosensors, in this review, we surveyed various electrical platforms that utilise nanoparticles and nanostructures as key materials for the detection of DA released from living cells (Table 1). Several excellent review articles on DA biosensors have already been published. However, given the growing interest in cell-based therapeutics and drug-screening tools to treat neurological diseases, a review focusing on DA detection in living cells is required. The main focus of this review was the functionalisation of the electrode surface that serves as both a signal acceptor and cell cultivation platform with various nanomaterials (e.g., carbon nanotubes, graphene derivatives, gold nanostructures, metal oxides, and polymers). Following the EC techniques, we have also summarised nanomaterial-modified FET-based DA sensors that may overcome the sensitivity and selectivity issues of EC biosensors. This review will accelerate research on the development of electrical biosensing platforms that enable rapid, sensitive, and selective detection of target molecules from living cells, ultimately contributing to the improvement of therapeutic materials and methods for curing various diseases/disorders (Fig. 1).

**Table 1:** Nanomaterial-modified electrochemical sensors for detection of DA in living cells

Materials	Methods	Cell line	Linear range [µM]	LOD [nM]	Ref.
Reduced graphene oxide, platinum nanoparticles	CV, DPV	PC 12	0.087–100	5	[51]
Nitrogen-doped mesoporous carbon nanosheets	CV, AM	PC 12	0.001–500	10	[52]
Reduced graphene oxide, Zn-NiAl layered double hydroxide	CV, DPV	SH-SY5Y	–	0.1	[53]
Carbon nanotube, Polypyrrole, sodium dodecyl sulphate	CV, AM DPV	PC 12	0.005–10	0.136	[54]
Carbon nanotube, AgAu nanoparticles	CV, AM	PC 12	0.003–2300	0.23	[55]
Carbon nanotube, Graphene quantum dots	CV, AM	PC 12	0.005–100	0.87	[56]
Carbon fibre, gold	CV, AM	PC 12	–	–	[57]
Nanocone-shaped 3D gold structures	CV, AM	PC 12	1–43	184	[58]
Micro pyramid-shaped 3D gold structures	CV, AM	SH-SY5Y	0.01–500	0.5 ± 0.08	[59]
FePt-Fe_3_O_4_ Nanoparticles	CV, AM, DPV	PC 12	0.005–0.11	1	[60]
Mesoporous Fe_3_O_4_	CV, AM, DPV	PC 12	0.002–0.6	0.8	[61]
Mesoporous ZnFe_2_O_4_	CV, AM, DPV	PC 12	0.002–0.6	0.4	[62]
MXene-micropatterned field-effect transistors	Conductivity variation, AM	Hippocampal neurons	50–2000	100	[63]
Nafion™ film-coated carbon nanotube-based field-effect transistors	Conductivity variation, AM	PC 12	0.01–100	10	[64]
Polypyrrole nanotube/Aptamer-based liquid-ion gated field-effect transistors	AM	PC 12	0.0001–s10	0.1	[65]

## Electrochemical detection of dopamine (DA) release from living cells

EC methods are powerful tools for creating sensitive and selective sensing platforms owing to several properties such as rapid detection time, convenience, and stability. To fabricate EC sensors to detect DA release in living cells, key factors need to be satisfied with the electrode surface materials. First, excellent electrical properties, including electrical conductivity and electron mobility, accelerate the redox reaction as a critical mechanism in EC sensing. Second, biocompatibility does not inhibit the adhesion, function, and growth of neuronal cells that adhere to the surface. In this section, we categorise the materials into three different areas: (i) Carbon-based materials, (ii) hybrid gold nanoparticles, and (iii) bare gold structures with different geometries.

### Carbon-based materials for the development of electrochemical dopamine sensors

Carbon nanomaterials, a class of materials mainly composed of carbon (i.e., the graphene family, CNTs, and carbon nanofibers (CNFs)), have been utilised in various fields due to their unique properties such as thermal stability, chemical resistance, and excellent mechanical properties [66–69]. Generally, most carbon-based materials have a specific chemical structure, where carbon atoms combine via SP^2^ hybridisation, which causes delocalisation of electrons in their structure [70]. Owing to their structural features, they have excellent electrical properties, including electrical conductivity and electron mobility. In addition, carbon-based nanomaterials with enhanced electrical ability and additional capabilities can be obtained through chemical or physical modification [71]. Given that biosensing based on EC detection techniques (i.e., cyclic voltammetry, amperometry, and differential pulse voltammetry) requires a platform with excellent electrical properties, carbon-based nanomaterials have tremendous potential for sensing target biomolecules with their redox activities.

#### Graphene family-based dopamine (DA) sensors

**Fig. 2:**
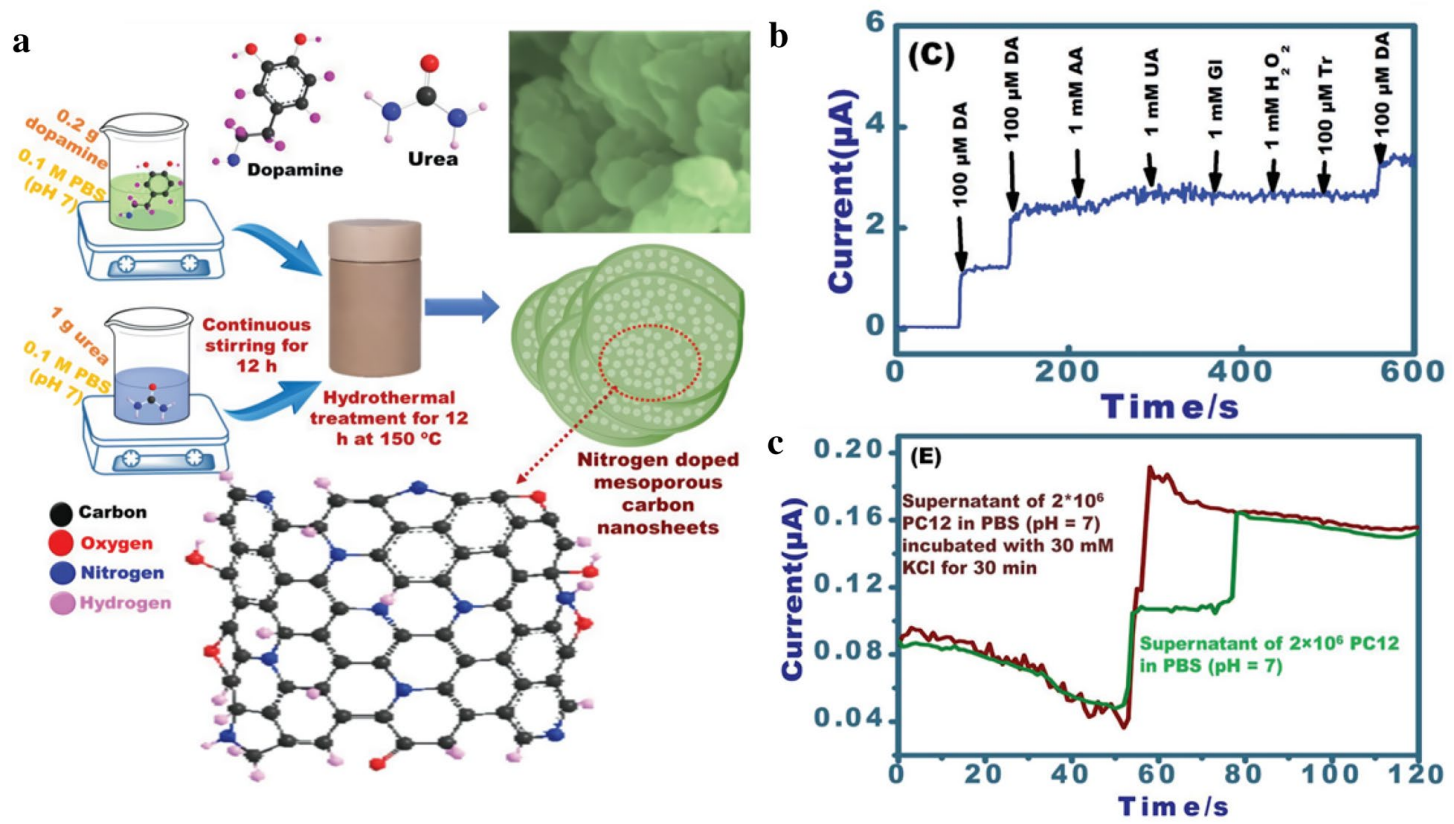
**a** Schematic illustration representing the 3D-N-doped mesoporous nanosheets-based DA sensing platform. **b** Amperometric responses of the platform showing its selectivity toward DA. **c** Amperometric data of DA secreted from PC12 cells with (brown graph) or without (green graph) stimulation of DA release, with permission from [52]; Copyright 2018 WILEY-VCH Verlag GmbH & Co. KGaA, Weinheim

To date, the graphene family has been widely used for the electrical detection of DA with the modification of other chemicals such as gold nanoparticles. In addition, graphene is controllable in terms of shape, size, and structure. Recently, Zan et al. reported that a reduced graphene oxide-based EC sensing platform is highly effective in detecting DA released from pheochromocytoma cells [51]. In this study, a reduced graphene oxide composite was fabricated with a flexible ultra-thin structure. Moreover, the graphene composite was modified with dendritic platinum nanoparticles, which increased the sensitivity of sensing DA in living cells. To fabricate the sensing platform, ultra-thin reduced graphene oxide was prepared by sequential mould casting, a method suitable for controlling the size and thickness of graphene and chemical reduction. Subsequently, the reduced graphene oxide composite was decorated with platinum nanoparticles synthesised by thermal synthesis through the dip-coating method. Based on the results of EC detection, such as differential pulse voltammetry (DPV) and cyclic voltammetry (CV), it was confirmed that this sensing platform could recognise EC signals from DA in the presence of interfering molecules (i.e., ascorbic acid and uric acid). Furthermore, it was demonstrated that the sensing platform is adequate for cell cultivation and the detection of DA secreted from living cells. In 2017, Emran et al. developed a novel carbon material-based amperometric sensing platform for monitoring DA from living cells (Fig. 2a) [52].

Interestingly, the carbon materials used in this study have unique structures and properties, specifically, three-dimensional N-doped mesoporous nanosheets with excellent biocompatibility. Owing to their structural and chemical features, the sensing platform has several advantages: (i) high surface area, (ii) excellent biocompatibility, and (iii) enhanced electrocatalytic features for highly sensitive detection of DA released from living cells, which leads to improved detection capability (a linear range of < 0.5 mM and a limit of detection (LOD) of 10 nM), as compared with that of conventional sensing electrodes. Moreover, the amperometric responses from chemical DA were selectively recognised in the presence of several interfering molecules (Fig. 2b). Above all, it was observed that DA from living cells was detectable under K + stimulation through amperometric responses (Fig. 2c). In 2019, Asif et al. introduced highly sensitive and selective DA sensors composed of hetero-stacked layers, including reduced graphene oxide (rGO) and layered double hydroxides (LDHs) [53]. Specifically, the LDHs improved the catalytic capability of the composite. Additionally, the rGO layer was hybridised with an extremely thin LDH layer. It was fabricated on a single molecular scale to improve both the surface area of the composite and the efficacy of electrocatalytic activity. According to the CV measurements, it was observed that the electrochemical peaks of ascorbic acid (AA), uric acid (UA), and DA could be separated in the presence of three chemicals. Thereafter, the LOD of the sensor was calculated through DPV analysis, with an LOD of DA of 0.1 nM. Moreover, the rGO-based composite for DA detection was utilised in the cultivation of neuroblastoma cells. In this study, it was demonstrated that the composite was biocompatible, and can identify the EC signals of DA released from living cells through DPV measurement.

#### Carbon nanotube (CNT)-based dopamine (DA) sensors

**Fig. 3:**
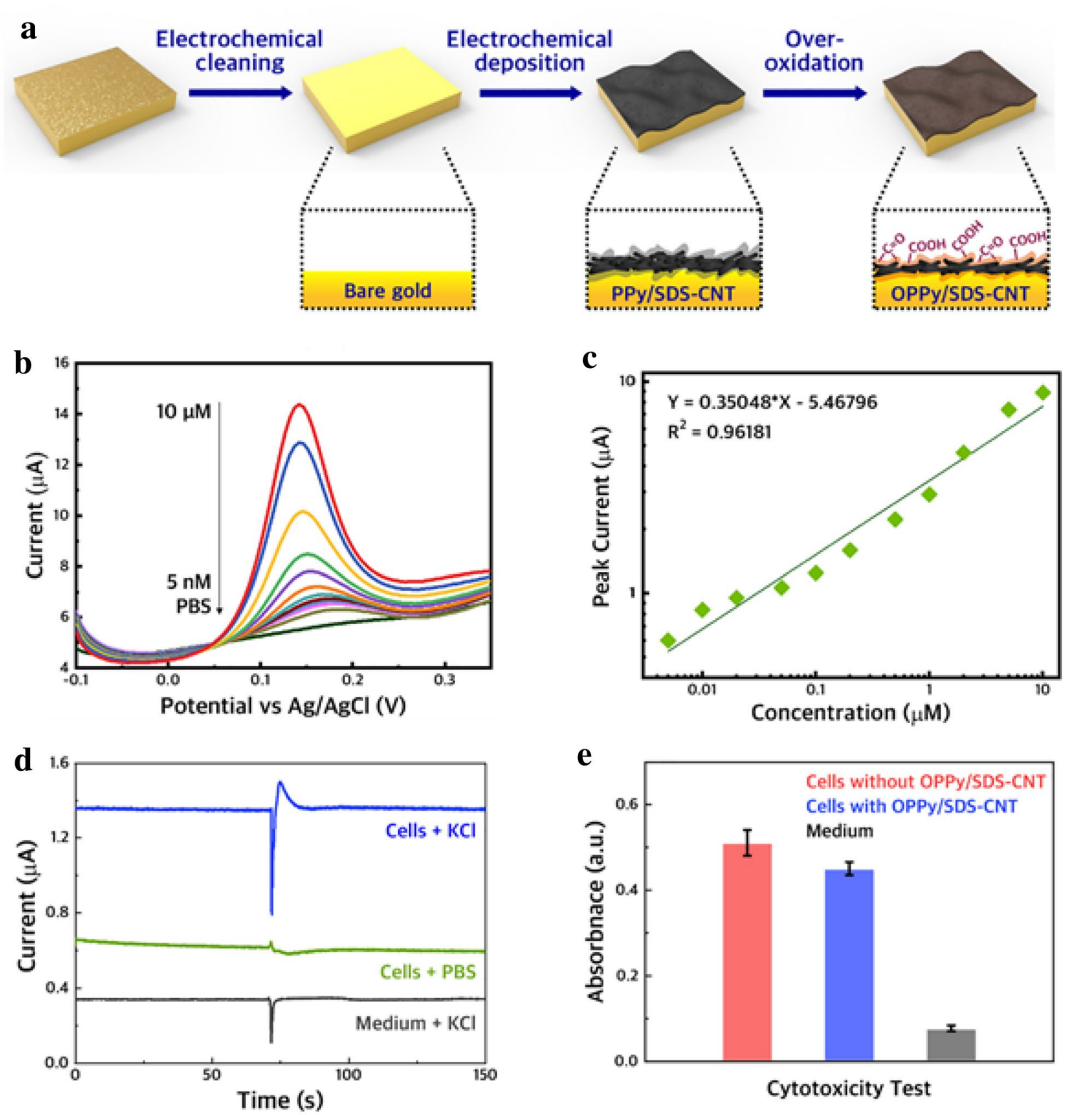
**a** Schematic illustration showing an oxidised polypyrrole/sodium dodecyl sulphate-modified multi-walled carbon nanotube (OPPy/SDS-CNT) electrode for the detection of DA. **b** DPV graphs of the OPPy/SDS-CNT electrode with several concentrations of DA (5 nM–10 µM). **c** Regression analysis of the current peaks obtained from **b**. **d** Amperometric currents of DA were released from cells using the OPPy/SDS-CNT electrode. **e** Cytotoxicity test of the OPPy/SDS-CNT electrode using the MTT assay. With permission from [54], Copyright 2019 Elsevier B.V

According to previous studies, 3D-carbon-based materials have outstanding electrical properties that function advantageously in electrical sensing [72]. For instance, multi-wall carbon nanotubes (MWCNTs), a 3D-carbon-based material with a cylindrical structure, have more than two times higher electrical conductivity than graphene [73]. Through the application of CNTs, Eom et al. reported a novel CNT-based sensing platform for the sensitive detection of DA (Fig. 3a) [54]. To fabricate the sensing platform, the CNTs were modified with overoxidised polypyrrole (OPPy), where its amine group increases the sensitivity and electroactivity of the sensing platform. According to the EC results, the platform exhibits excellent selectivity in the presence of interfering molecules (i.e., ascorbic acid and glucose), and the LOD of the platform was 136 pM (Fig. 3b, c). Additionally, the platform was utilised for the cultivation of dopaminergic neurons. During cultivation, it was confirmed that the platform is biocompatible and a specific amperometric current of DA released from the cells is detectable through EC measurement.

In another study, MWCNTs and AgAu nanoparticle-based electrodes as DA sensors for monitoring DA release have been reported [55]. Interestingly, the MWCNTs used in the sensor were modified with AgAu nanoparticles. The AgAu nanoparticles, as a noble metal, increased the electron transfer rate and enhanced the electrochemical performance of the bare MWCNTs. In addition, the electrochemical sensor was quantified using CV analysis, and its LOD and linear range were 0.23 nM and 3 nM–2.3 mM, respectively. Above all, it was demonstrated that extracellular DA could be monitored in PC12 cells cultured on the sensor. In 2020, a novel MWCNT and graphene quantum dot-based electrochemical sensor to detect extracellular DA was reported [56]. Since the graphene quantum dots used in the modification of MWCNTs increased the surface area of the sensor, the electrical properties of the MWCNTs were improved. Based on the CV results, the LOD of the sensor was calculated as 0.87 nM with a wide linear range of 0.005 to 100 µM. Moreover, the sensor showed good cell biocompatibility through the cell viability test. Above all, the sensor could detect a specific EC DA peak, and the sensor could discriminate the level of DA release induction by KCl.

Taken together, it can be concluded that carbon materials have various advantages in sensing techniques. To be specific, it has been proven that carbon materials possess biocompatible properties and excellent EC features. Furthermore, carbon-materials are readily manufactured using various nanostructures. For these reasons, carbon-materials are outstanding candidates for the detection of DA.

### Gold nanostructure-modified electrochemical dopamine sensors

Gold nanoparticles are an advantageous material for biosensing, owing to several properties such as excellent conductivity, signal amplification, and good biocompatibility [74]. In particular, gold nanoparticles have been utilised in various types of biosensors, including localised surface plasmon resonance sensors, optical sensors, and EC sensors [32, 75, 76]. In particular, various EC sensors for the detection of DA released from cells have recently been developed, as gold can enhance cell adhesion and growth and has outstanding electrical properties [77]. In terms of structures, there are three strategies to utilise gold to detect DA released from cells.

#### Electrochemical dopamine sensors modified with various bare gold structures

In 2019, a new 3D-nanoelectrode modified with gold to detect DA from living cells was devised [58]. In particular, nanoelectrodes have a specific nanocone-shaped 3D structure. To fabricate the gold nanoelectrode, glass capillaries were prepared using laser pulling, and then gold was coated onto the surface of the treated glass electrode. CV confirmed that the gold nanoelectrode showed higher EC peaks toward DA than the bare nanoelectrode. Moreover, the current responses of DA exocytosis from single cells were observed when the gold nanoelectrode was close to the cell.

**Fig. 4:**
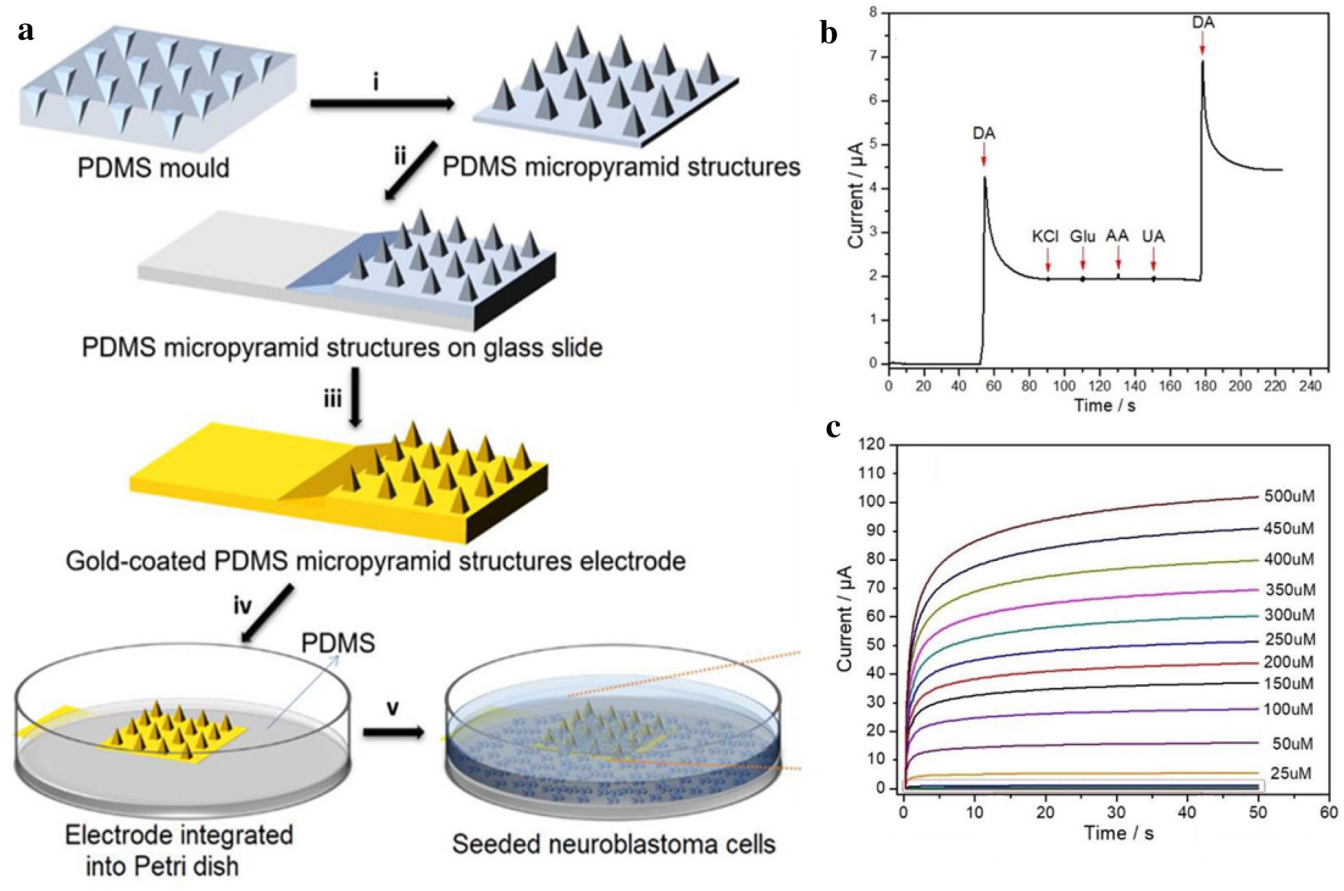
**a** Schematic illustration of the fabrication processes for micro pyramid platform structures. **b** Selectivity test of dopamine with interfering substances. **c** The amperometric responses of micro pyramid platform with several concentrations of DA (500 µM–10 nM). With permission from [59], Copyright 2020, Analytical chemistry

In 2018, Kim et al. also showed 3D-structural gold nanopillar pattern arrays to detect DA [78]. In this study, laser interference lithography was used to fabricate highly homogeneous polymer nanohole patterns. Subsequently, the EC deposition technique was used to deposit gold into the nanohole patterns, and then, the polymer nanohole patterns were sensitively removed to achieve 3D-structural gold nanopillar pattern arrays. Through CV and amperometry, the platform showed excellent sensing capabilities, with a detection limit of 5.83 µM, and DA was detectable in the presence of interferants. Thus, the gold 3D structure of this study was sensitive to DA because of its high surface area and conductivity. In summary, gold-based 3D structures and highly sensitive DA sensors with high surface areas can be fabricated using electrodeposition techniques. This approach has been utilised to fabricate 3D-structural electrochemical sensors for detection of DA released from living cells in many studies. Recently, a study by Senel et al. used a gold-deposited EC platform on which countless micro-sized pyramid models were fabricated to increase the surface area to be arrayed to effectively detect DA secreted from neuroblastoma cells by EC methods [59]. The sensing electrode was fabricated by incubation, salinisation, and EC deposition, as illustrated in Fig. 4a. The sensing electrode has excellent sensitivity against DA (Fig. 4b). The neuroblastoma cells cultured on the fabricated platform showed excellent potential for the electrocatalytic activity to release DA. The LOD was 0.5 ± 0.01 nM, and the wide linear range was from 10 nM to 500 µM (Fig. 4c). These parameters offer a significant advantage in that real-time analyses directly from living cells can be achieved by measuring the released DA by culturing the neuroblastoma cell on the fabricated platform. Thus, this system also offers a significant advantage owing to its high selectivity and large surface area achieved through surface modification and can recognise the DA secreted from living cells.

Taken together, gold is an outstanding material for the fabrication of DA sensors owing to its high biocompatibility and conductivity, which contribute to the improvement of sensitivity of DA detection from living cells.

#### Hybrid gold nanoparticle-based electrochemical dopamine sensors

**Fig. 5:**
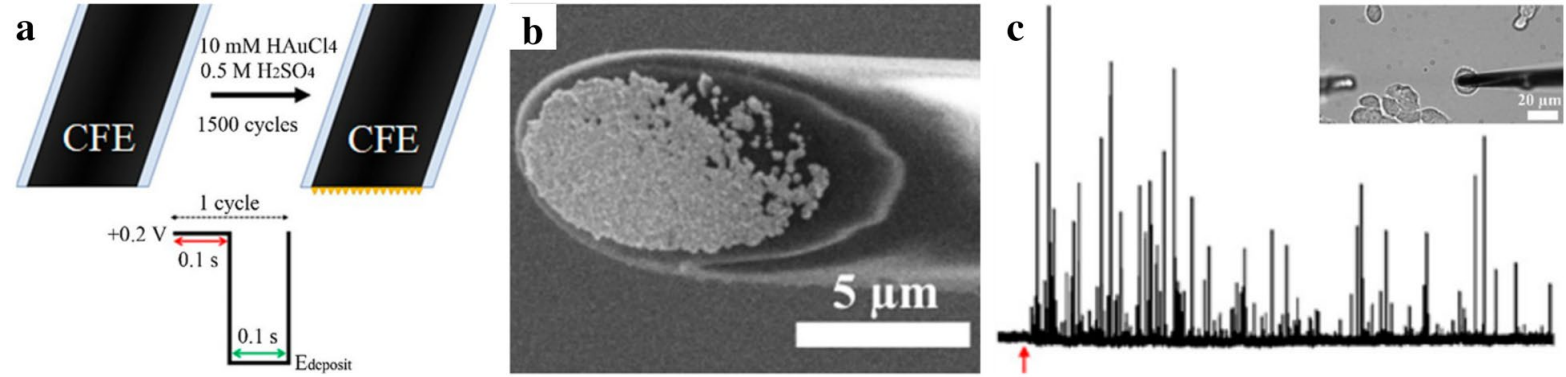
**a** Schematic illustration showing fabrication processes of GNP-modified carbon fibre electrode. **b** Surface electron microscope image of GNP-modified carbon fibre electrode. **c** Amperometric monitoring of DA released from single PC12 cell using the GNP-modified carbon fibre electrode. The microscopic image of a single PC12 cell during amperometric detection is shown in the inset image. The red arrow represents initiation point of stimulation. With permission from [57], Copyright 2018, American Chemical Society

The first strategy is to modify existing electrodes with gold nanoparticles (GNPs), which have been used as an important material for the fabrication of DA sensors owing to several properties, such as good chemical stability and electrical conductivity. Furthermore, GNPs have rich surface chemistry, which can be easily conjugated with other chemicals [79]. Several studies have demonstrated the attractiveness of GNPs as DA sensors. In 2019, a novel gold electrode was modified with GNPs, Nafion™ (NF), and β-cyclodextrin (CD) for EC detection of DA [80]. Specifically, the GNPs were electrodeposited onto the surface of the carbon fibre electrode. According to the cyclic voltammograms, the EC peaks of DA were higher at the GNP-modified gold electrode than at the bare gold electrode, indicating that GNPs functioned as an EC signal enhancer. In addition, the highest EC peaks of DA were obtained at the GNP/NF/CD-modified gold electrode, which showed the synergistic effect of the combination of GNPs and the chemicals on DA sensing. Moreover, DA diluted in urine was detectable using the GNP/NF/CD-modified gold electrode for DPV measurement, which demonstrated that the GNP-based DA sensor can detect DA sensitively in the presence of many interfering molecules. In summary, GNPs are useful materials for the EC detection of DA. In 2018, a carbon fibre electrode, an existing electrode modified with GNPs for detecting DA released from living cells, was reported (Fig. 5a) [57]. To fabricate the GNP-modified carbon fibre electrodes, the GNPs were electrodeposited onto the surface of the carbon fibre electrodes (Fig. 5b). The modified electrodes showed higher EC DA peaks than that of the bare carbon fibre electrode, which indicates that GNPs increased the electron transfer during DA detection and eventually improved the sensitivity of the DA sensor. Furthermore, the levels of DA released from pheochromocytoma cells (PC12) were monitored successfully using the modified electrode through amperometry (Fig. 5c).

### Metal oxide-based dopamine (DA) sensors

With the rise of the industrial revolution, researchers have begun to study how to store large amounts of energy [81] efficiently. Batteries made from metal oxide are widely used in mobile phones, laptop computers, among other applications. In particular, metal oxides in nanomaterials are easy to manufacture on a large scale and have highly specific capacities due to abundant redox reactions containing various ions [81]. In this section, we highlighted recent studies using metal oxide nanoparticles and nanostructures as the core material to enhance the EC signals of DA, as well as to improve the biocompatibility of the electrodes.

#### Morphologically transformed metal oxide-based dopamine (DA) sensors

Specifically, morphologically transformed iron oxide nanoparticles remain an issue for detecting nanomolar units of dopamine under physiological conditions but possess excellent electrolytic properties; hence, they are applied to these iron oxides with electrodes. In 2017, Yang. W. et al. synthesised iron oxides and platinum to create a FePt-Fe_3_O_4_ dumbbell-like nanostructure and measured dopamine [60]. The electrode was fabricated by repeated heating and cooling, and the disposal-precipitation processes were repeated. The LOD of the generated platform for DA was 1 nM, and the linear range was 0.1 µM to 90 µM. In addition, selectivity was demonstrated via EC analysis of DA in the presence of 1.0 µM uric acid, 1.0 µM ascorbic acid, and 0.1 µM dopamine. After growing PC12 cells on the platform and stimulating the cells with KCl solution to induce DA release, the electrode recognised released DA, an indication of biocompatibility.

In addition to iron oxide, researchers have investigated other oxides such as nickel oxide, which has excellent electrochemical performance at a low cost to the electrodes. It is environmentally friendly and is well known for its unique and attractive properties as it is used as an excellent electrode material for pseudocapacitors [82]. In 2018, Emran et al. developed a metal oxide nanomaterial platform based on lacy flower-like NiO. The sensor was fabricated by ultrasonication and deposition on an ITO electrode [83]. The detection limit was 85 nM, and the linear range was 0.5 µM to 5 µM. After the PC12 cells were cultured, the supernatant was isolated and measured while simultaneously measuring the isolated supernatant. As the supernatant of PC12 cells was added, the amperometry current was observed to change. This finding indicated that the fabricated sensor has biocompatibility and allows real-time measurement with amperometry because of its ability to measure secreted DA.

**Fig. 6:**
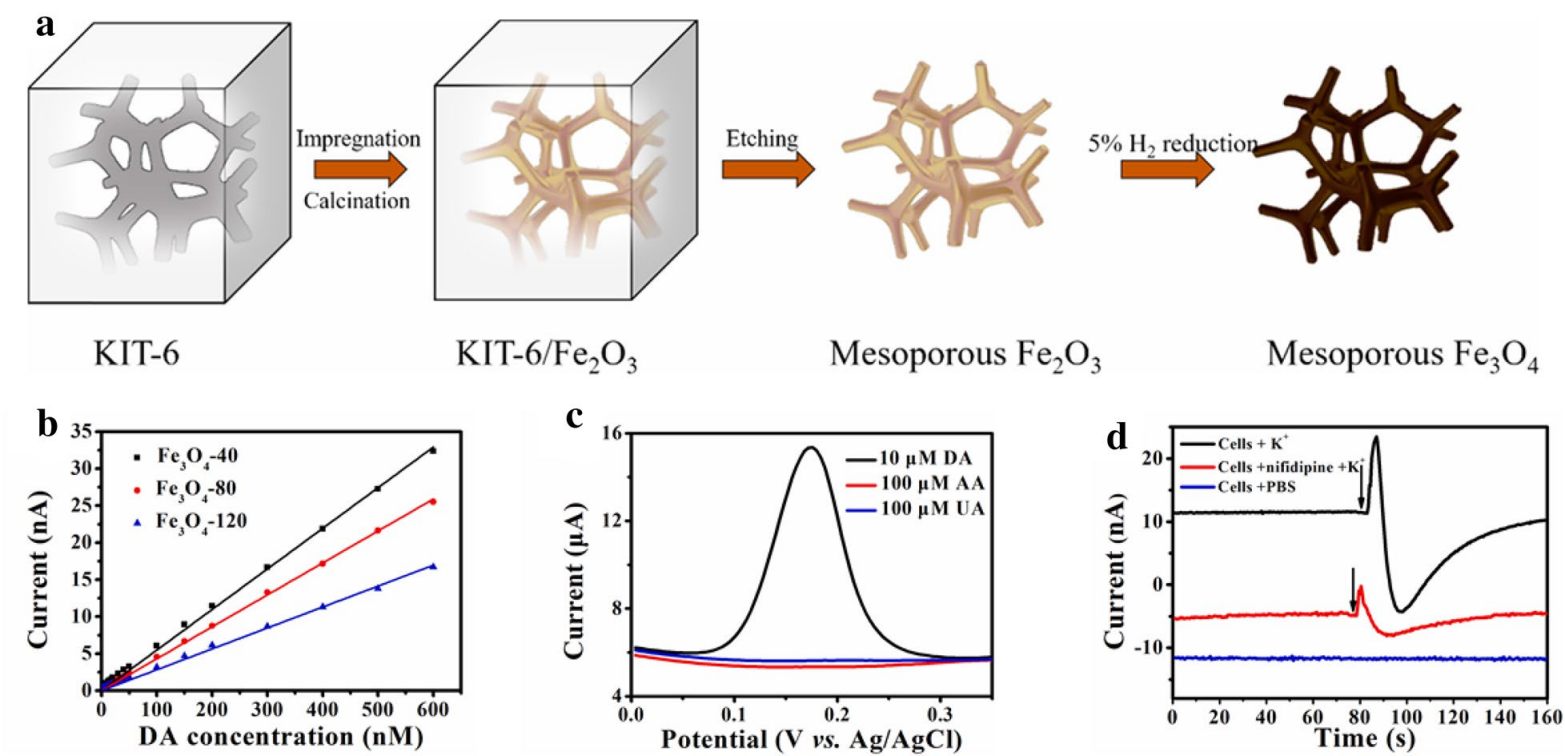
**a** Schematic illustration of electrode fabrication. **b** The plot of fabricated electrodes with various concentrations of dopamine. **c** Selectivity test of dopamine against interfering substances. **d** The current change test with KCl stimulation (arrow points) of PC12 cells. With permission from [61]; Copyright 2019, Elsevier

#### Mesoporous metal oxide-based dopamine (DA) sensors

Some metal oxides with generalised structures have the disadvantage of low conductivity [84]. For this reason, research has begun to actively detect analytes by modifying the state of the metal oxide. Typically, attempts are made to transform a metal oxide structure into a mesoporous form because this structure has excellent topological stability, controllability, and veracity. In 2019, an EC platform with a mesoporous design was reported [61]. The scaffolds were fabricated by impregnation, calcination, or etching, and finally, a mesoporous structure was achieved (Fig. 6). Of these electrodes, the best detection limit was 0.8 nM, and the linear range was between 2 and 600 nM. In the presence of ascorbic acid and uric acid, the sensor showed excellent selectivity regardless of the DA detection ability. The DA released from cultured PC12 cells was measured in real-time using amperometry, and changes in current were observed (Fig. 6d). By monitoring the DA released from living cells in real-time using amperometry, these electrodes were shown to be excellent sensors with high biocompatibility, selectivity, and sensitivity.

#### MFe_2_O_4_-based dopamine (DA) sensors

Recently, research has been conducted to create a series of MFe_2_O_4_ (M = Ni^2+^, Mn^2+^, Fe^2+^) structures by attaching different metal structures to iron oxide [85]. In particular, ZnFe_2_O_4_ has received attention as an excellent material for electrode applications because it is non-toxic, odourless, biocompatible, acid- and alkali-insoluble, and is stable at high temperatures [86]. In 2020, Huang et al. developed a platform based on ZnFe_2_O_4_ with mesopores [62]. The platform was fabricated by the hitting method, and showed excellent performance, with a detection limit of 0.4 nM and a linear range of 2–600 nM. The presence of interfering substances such as a tenfold concentration of NaCl, uric acid, and ascorbic acid does not interfere with DA detection, indicating that the fabricated EC platform has excellent selectivity for dopamine. Owing to such excellence in the sensor performance, dopamine exocytosis from PC12 cells was also successfully measured by amperometry in real-time under K^+^ stimulation condition. To increase the EC detection efficiency, it is critical to add or synthesise a material for application to the electrode.

As a short summary of EC biosensors, the capacity for detecting an analyte varies depending on the electrode structural design or fabrication methods. By applying these features, research is continually progressing, and the sensitivity and selectivity toward the analyte can be achieved with maximum efficiency. Therefore, it is expected that an electrode with excellent detection capacity will be developed soon.

## Field-effect transistors (FET) for the detection of dopamine (DA) released from living cells

Field-effect transistors (FETs) are the type of transistors that operate based on the flow of current, which is controllable by the voltage applied. Unlike the EC sensors that are composed of working, auxiliary, and reference electrodes, FETs are composed of source, drain, and gate, wherein either electrons or holes exist to control the conductivity of the channel. The electrical signals of FETs are generally expressed as the changes in current intensities and are proven to be extremely sensitive to the alterations of the external environment. Even though the fabrication process is quite complex, this extreme sensitivity made the FETs become one of the most popular platforms in the biosensor area [87, 88]. Since the amount of DA exocytosed from neuronal cells is extremely low, this advantage of FETs, that is, the high sensitivity, is preferred to construct a DA biosensor. In this section, we will highlight recent studies using FET as a core-sensing platform to detect dopamine from living neurons, with particular focus on the use of various nanomaterials including MXenes, CNTs, and conductive polymers to improve both sensitivity and selectivity.

###  Nanocomposite integrated field-effect transistor (FET)-based dopamine (DA) sensors

Since 2011, MXene has been in the spotlight in the electrical, chemical, and physical fields, as it has shown excellent properties in various areas, including energy storage, display, and biosensor. In 2016, there was a new attempt to sensitively detect dopamine using FET-MXene to investigate cell function in a biologically relevant event [63]. The platform was fabricated by selective etching and printing using the microcontact method. The detection limit was 0.1 µM, and the linear range was from 0.1 µM to 50 µM. The neuronal hippocampal cells were cultured, and the cells were seeded on the FET sensor. Depending on the K + concentration, the neuronal cell reaction was immediately monitored, which indicated that the fabricated sensor had low cytotoxicity to living cells and high biocompatibility. As such, this experiment demonstrates the meaning of performing quantitative and real-time evaluation by applying FET to cells.

**Fig. 7:**
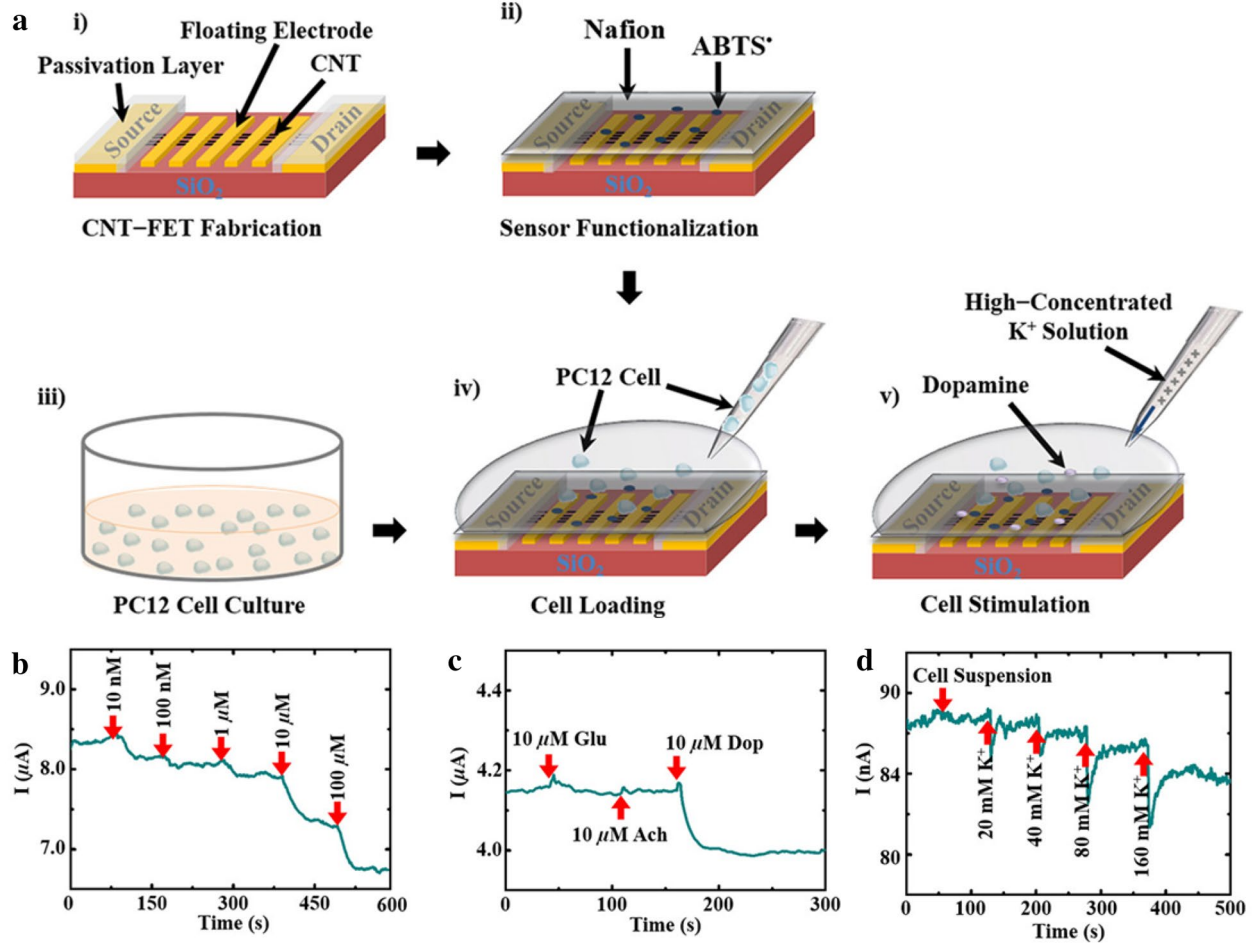
**a** Schematic illustration of the platform fabrication and culturing of PC12 cells. **b** The real-time amperometry test for dopamine. **c** The dopamine selectivity test for the fabricated platform. **d** The real-time amperometry test for current changes with K^+^ stimulation of a cell suspension solution. With permission from [64]; Copyright 2019, ACS applied materials and interfaces

In addition to MXenes, CNTs were also attempted to be integrated with FET device owing to the several advantages of CNTs including the robustness, high conductivity, and high affinity toward biomolecules [89]. Pham et al. introduced a Nafion™-radical on a FET to create a platform that can sense DA (Fig. 7) [64]. The platform was fabricated through ultrasonication, thermal evaporation, and a lift-off process. The fabricated platform has an excellent LOD and linear range of 10 nM and 1 nM–100 µM, respectively. Acetylcholine and glutamate were added concurrently to investigate selectivity while measuring the DA secreted from PC12 cells. There was a change in electrical current by DA secretion; however, no significant current change was observed with the interfering substances. Because the PC12 cell is placed on the sensor, and dopamine can be measured, this sensor has the advantage of measuring dopamine in living cells and can be observed in real-time. As a result, by bonding the material to FET, the mobility of electrons and the affinity between the analyte and the electrode was increased, thereby providing a foundation for this method to have higher selectivity and sensitivity.

### Conductive polymer integrated field-effect transistor (FET)-based dopamine (DA) sensors

**Fig. 8:**
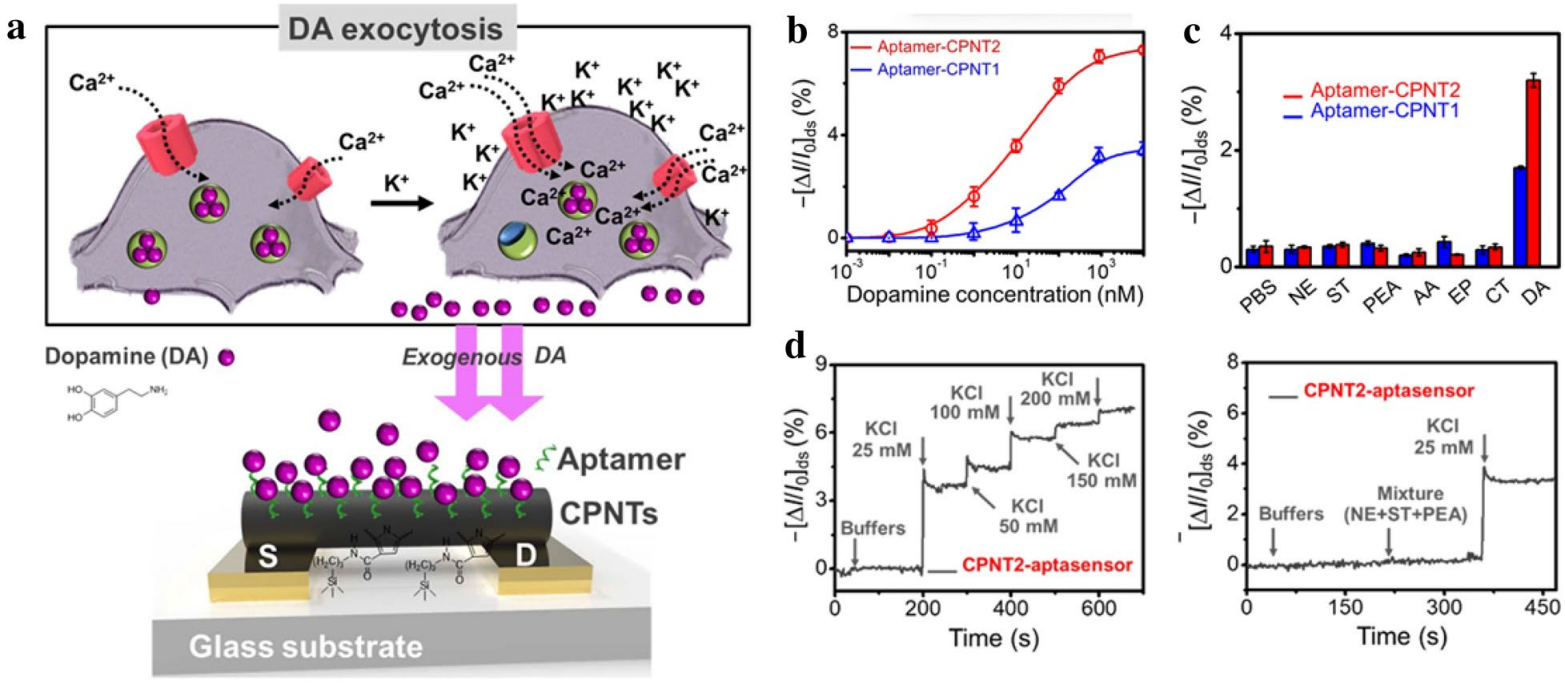
**a** Schematic illustration representing the carboxylated polypyrrole nanotubes (CPNTs)/Aptamer-functionalised liquid-ion gated field-effect transistor (FET)-based DA sensor. **b** Amperometric test of various concentrations of dopamine with two types of tubular structured-aptasensors (CPNT1 and CPNT2). **c** Selectivity analysis of the CPNTs/aptamer-based sensor against interfering substances. **d** The amperometric response of the CPNTs/aptamer-based sensor for monitoring dopamine exocytosis at different KCl concentrations. With permission from [65]; Copyright 2020, Scientific reports

Since polyaniline, polythiophene, and polypyrrole (PPY) are conductive polymers that generally have a chain structure, they have excellent electron mobility and can efficiently transfer electrical signals. Conductive polymers can have synergistic effects with FETs and have advantages such as solution process ability and tunable properties [90]. Using such compounds, the detection of DA secreted from cells using PPY has been reported. In 2020, a study by Park et al. measured DA from living cells using FET [65]. Nanotubes with carboxylated polypyrrole sensors were fabricated using a chemical conjugation method with an aptamer designed for specific interaction with dopamine (Fig. 8). The detection limit was as low as 100 pM, and the linear range was from 0.1 nM to 10 µM. Furthermore, catechol, epinephrine, and ascorbic acid did not interfere with DA sensing. After culturing the cells, the released dopamine was measured with the sensor, and it was confirmed that the fabricated platform has a biocompatibility. This result suggests that recognising DA exocytosis in living cells is possible and thus improves the role of the platform as a biosensor for in vitro experiments. Application of FET to a sensor offers the advantage of improved sensitivity over existing electrochemical sensors. FET applications offer DA detection the possibility of sensing with high sensitivity, which is the basis for developing a sensitive sensor that can evolve into later generations.

## Conclusion and future perspectives

In this review, we summarised recent studies of nanomaterial-modified electrical platforms for the detection of dopamine from living cells. Various nanomaterials (e.g., carbon-based materials, gold, metal oxide, and conductive polymers) were reported to be excellent for improving both the sensitivity and selectivity of electrical platforms for exocytotic dopamine detection. Specifically, in the case of electrochemical detection, the nanomaterials can facilitate electron transfer reactions on the electrode surface and adjust the redox state of the sample, improving the detection of unique peaks, currents, and potentials. For FET-based sensors, various nanomaterials including MXenes, CNTs, and conductive polymers were incorporated to immobilise the bioligands (e.g., antibodies and aptamers) and to sensitise the electrical properties of the sensor surface wherein the binding reaction between DA and bioligand occurs.

Unlike typical in vitro DA detection, the DA sensor specialised for living neurons should satisfy biocompatibility. As aforementioned, the nanomaterials can improve the electrical properties for both electrochemical and FET sensing platform; however, the diversity of nanomaterials is limited since the living neuronal cell should attach and grow on the sensor surface. The adaptation of several cytophilic materials, including ECM (extracellular matrix) proteins (e.g., fibronectin, laminin, and vitronectin), collagens, and peptides, can be an excellent candidate to overcome the biocompatibility issues of metallic materials, including silver nanoparticles and nanostructure, which is highly conductive but is toxic to cells.

As a short conclusion, the electrical biosensors capable of measuring DA from living neurons will facilitate the discovery of new types of drugs and techniques, which will ultimately contribute to treatment of various DA-related diseases, including Parkinson’s, schizophrenia, and Huntington.

## Data Availability

The datasets used and analysed in this study are available from the corresponding author upon reasonable request.

## Abbreviations

AM: Amperometry
AA: Ascorbic acid
ABTS: 2,2′-Azino-bis(3-ethylbenzothiazoline-6-sulfonic acid)
CFE: Carbon fibre electrode
CNT: Carbon nanotube
CPNTs: Carboxylatedpolypyrrole nanotubes
CD: β-Cyclodextrin
CV: Cyclic voltammetry
DPV: Differential pulse voltammetry
DA: Dopamine
ECM: Extracellular matrix
EC: Electrochemical
ECL: Electrochemiluminescence
FETs: Field-effect transistors
GNPs: Gold nanoparticles
HD: Huntington's disease
LDHs: Layered double hydroxides
LOD: Limit of detection
MWCNTs: Multi-wall carbon nanotubes
NF: Nafion™
PPY: Polypyrrole
OPPy/SDS-CNT: Oxidised polypyrrole/sodium dodecyl sulphate-modified multi-walled carbon nanotube
PD: Parkinson's disease
PC12: Pheochromocytoma cells
rGO: Reduced graphene oxide
UA: Uric acid
